# Awareness of Endometriosis Symptoms—A Cross Sectional Survey among Polish Women

**DOI:** 10.3390/ijerph18189919

**Published:** 2021-09-21

**Authors:** Maja Kotowska, Justyna Urbaniak, Wojciech J. Falęcki, Piotr Łazarewicz, Mateusz Masiak, Iwona Szymusik

**Affiliations:** 1 Students Scientific Association at the 1st Department of Obstetrics and Gynecology, Medical University of Warsaw, 02-015 Warsaw, Poland; kotowska.maja@gmail.com (M.K.); justyna.urbaniak7@gmail.com (J.U.); piotrek@lazarewicz.eu (P.Ł.); 2 1st Department of Obstetrics and Gynecology, Medical University of Warsaw, 02-015 Warsaw, Poland; wojciechjfalecki@gmail.com; 3 Calmsie, 20-060 Lublin, Poland; mateusz.masiak@calmsie.ai

**Keywords:** endometriosis, dysmenorrhea, pelvic pain, infertility, awareness, public health

## Abstract

*Background*: Endometriosis is a chronic inflammatory condition characterized by the presence of endometrial-like tissue outside of the uterine cavity. It affects approximately 6–12% among reproductive-age women. Laparoscopy is still the gold standard for diagnosing it. Since an average of couple of years elapses from the onset of symptoms to the definitive diagnosis, analysis of symptoms could serve as a non-invasive diagnostic tool. *Methods*: An anonymous survey was distributed online in November 2019. It contained 23 closed questions, which covered two areas: knowledge and awareness of endometriosis symptoms and its presence among respondents. *Results:* A total of 3319 women completed the survey, of which 328 were diagnosed with endometriosis (9.9%). The symptoms of endometriosis most often indicated by the general population were infertility and dysmenorrhea. In contrast, those least frequently indicated were painful defecation and dysuria. Respondents suffering from endometriosis indicated that they often or sometimes experienced dysmenorrhea (83%, *n* = 273), pelvic pain not related to menstruation (72%, *n* = 235), dyspareunia (68%, *n* = 223) and painful defecation or dysuria (40%, *n* = 132). *Conclusions:* Since the search for a non-invasive diagnostic endometriosis marker has been conducted for many years without success so far, it seems that awareness of the clinical presentation and reported symptoms may help to provide earlier diagnoses.

## 1. Introduction

Endometriosis is a chronic inflammatory condition, characterized by the presence of endometrial-like tissue outside of the uterine cavity. It is estimated that the global incidence of the disease is approximately 6–12% among reproductive-age women [1]. Symptoms that include dysmenorrhea, dyspareunia, non-menstrual pelvic pain, nerve pain and infertility vary among patients and may not correlate with disease severity [2]. Furthermore, patients with endometriosis are at higher risk of autoimmune, cardiovascular and psychiatric comorbidities [3,4,5]. The disease adversely affects the quality of life (QoL), bringing physical, social, emotional and professional burdens [6]. A study among 193 patients showed that due to symptom exacerbation, patients lost about one working day per week (an average of 7.4 h), 64% reported loss of productivity at work and 60% impaired daily functioning [7].

Currently, laparoscopic visualization followed by histopathologic confirmation is the gold standard for definitive diagnosis [8]. However, this invasive procedure generates high costs for the healthcare system and carries a risk of complications for the patients. The time between the onset of symptoms and diagnosis is reported to be between 2 and 12 years [9,10]. The prolongation factors include underestimation of symptoms by physicians, lack of widely available low-cost diagnostic tools and limited awareness of patients and general practitioners (GPs) about endometriosis.

For years, a non-invasive, validated diagnostic tool has been sought. It is believed that symptoms could be an important diagnostic tool. Awareness of the disease symptoms will contribute to earlier appointments with specialists, and thus earlier implementation of the appropriate treatment. The aim of our study was to evaluate women’s knowledge of endometriosis symptoms.

## 2. Material and Methods

The study was conducted using the computer assisted web interview (CAWI) method based on an original, anonymous questionnaire. The questionnaire was written in Polish and addressed to Polish speaking women only. It was prepared with the use of Google Forms tool and placed on popular feminine social media platforms in November 2019. The survey contained 23 close-ended questions, which covered two areas: (1) knowledge and awareness of endometriosis symptoms and (2) its presence among the respondents. The subgroup analyses were conducted on the basis of age, place of residence, education, medical/non-medical profession and being diagnosed with endometriosis (DxE). The latter group consisted only of those respondents who claimed to have received a diagnosis from a doctor based on ultrasonography or laparoscopy.

Nominal scales and a five-point Likert scale were used in the survey. The five-point scale assumed that the respondent indicated the following statements by marking the numbers, as below:

- “5”—strongly associates the symptom with endometriosis.
- “4”—moderately associates the symptom with endometriosis.
- “3”—neutral or not sure.
- “2”—does not associate the symptom with endometriosis.
- “1”—strongly believes it is not a symptom of endometriosis.

The online survey allowed for wide distribution of the questionnaire and obtaining a representative sample. The statistical analyses were performed using STATISTICA software v.14.0 (TIBICO Statistica, Palo Alto, CA, US). *p*- value < 0.05 was considered significant.

## 3. Results

A total of 3319 women completed the survey. Baseline characteristics of the study population are illustrated in Table 1.

In total, 95.7% of respondents were familiar with the term “endometriosis”; 92.9% of women indicated that endometriosis is a chronic disease; 84.2% were aware of its negative impact on QoL; and 36.8% correctly identified the prevalence of this condition.

The symptoms of endometriosis most often indicated by the general population were infertility and dysmenorrhea (75.8% and 68.1% indicated “5,” respectively). Dysuria and painful defecation were not perceived as symptoms of endometriosis by the majority: 1, meaning “I strongly believe it is not a symptom of endometriosis,” was given by 38.7% and 42.9%, respectively.

Women indicated: the Internet (90.5%), physicians (57.9%), medical articles (32.3%) and friends and relatives (25.8%) as the best sources of obtaining information on medical issues regarding endometriosis. Ninety-five-point-six percent of participants claimed that this issue is not covered enough and should be discussed more often in the media.

### 3.1. Respondents with Medical Occupations

Respondents with medical occupations accounted for 16.6% (*n* = 548) of the study’s population. This group of women identified eight symptoms of endometriosis more correctly than respondents without medical occupations. The above differences were statistically significant for all except dysmenorrhea. Painful defecation, sciatica and dysuria were the least known the manifestations of endometriosis. Table 2 shows a comparison of symptom identification by women with and without medical occupations.

### 3.2. Age Groups

The average age of the study group was 29.2 years (median: 29; minimum: 13; maximum: 63). Infertility and dysuria were symptoms most frequently indicated as strongly associated with endometriosis by respondents between 31 and 35 years of age; sacral pain and sciatica by women over 41; and diarrhea/constipation by the group between 36 and 40. All the above differences were statistically significant.

### 3.3. Education

All the symptoms were better identified by respondents with higher education in comparison to those with secondary or primary school education. However, the differences were statistically significant only for infertility (78.9% vs. 69.3% vs. 61.0% respectively; *p* < 0.0001), dyspareunia (34.6% vs. 31.2% vs. 25.0% respectively; *p* < 0.0001) and dysmenorrhea (71.2% vs. 62.3% vs. 47.0% respectively; *p* < 0.0001).

### 3.4. Place of Residence

Subgroup analyses by place of residence showed no significant differences between respondents living in large, medium or small cities or villages.

### 3.5. Respondents with Endometriosis

Respondents with endometriosis (DxE) made up 9.9% of the study population (*n* = 328). Mean time from the onset of symptoms to definitive diagnosis was 5.7 years (median 5; minimum: 1; maximum: 30). This subpopulation more often correctly identified all symptoms of endometriosis compared to respondents without endometriosis. The differences were statistically significant for all symptoms (*p* < 0.0001). Table 3 illustrates the identification of endometriosis symptoms by respondents with endometriosis (DxE) and without (Non-DxE).

### 3.6. Numbers of 4s and 5s Indicated

For each of the nine symptoms listed in the survey, women indicated 4 or 5 when they believed the symptom was a manifestation of endometriosis. We analyzed the number of respondents that indicated those values. Of all nine symptoms mentioned in the questionnaire, women with endometriosis properly identified eight or nine of them more often than women without endometriosis (32.6% vs. 7.8% respectively; *p* < 0.0001). The same analysis performed in the subgroups of medical occupation or not showed that women with medical occupations were more likely to recognize eight or nine of all symptoms (14.6% vs. 9.5% respectively; *p* < 0.0001).

### 3.7. Presence of Endometriosis Symptoms and Their Frequencies among the General Studied Population

Respondents were asked about the presence of four common symptoms of endometriosis (dysmenorrhea, chronic pelvic pain not related to menstruation dyspareunia, dysuria/painful defecation) and the frequencies of their occurrence. Respondents with endometriosis indicated that often or sometimes they experienced dysmenorrhea (83.3%, *n* = 273), chronic pelvic pain not related to menstruation (71.7%, *n* = 235), dyspareunia (68.0%, *n* = 223) and painful defecation or dysuria (40.3%, *n* = 132). They were more likely to report all of the above symptoms than women without endometriosis, and those differences were statistically significant.

## 4. Discussion

To the best of our knowledge, this was the first population-based attempt to assess the awareness of endometriosis symptoms. In 2017, scientists indicated top priorities for endometriosis research in the coming years. The development of a non-invasive screening tool to aid the diagnosis of endometriosis was at the forefront [11]. Symptoms of endometriosis are not characteristic, sometimes embarrassing and are often perceived by women and GPs as “typical menstrual complaints.” Moreover, a definitive diagnosis requires laparoscopy. This makes endometriosis a major social healthcare problem. According to the majority of our study population (84.2%), endometriosis strongly impairs the quality of life. This area has already been widely studied and addressed by many researchers. Moradi et al. presented the influences of endometriosis on various aspects of life, depending on age. For the youngest group of respondents (16–24 years) the disease had the greatest impacts on social life and education; for the group between 25 and 34 years of age the disease had the greatest impacts on life opportunities, employment and marital/sexual relationship; and for the oldest subgroup (35 and above) it had financial and physical impacts [12]. Patients with endometriosis also reported a negative influence on their professional development. De Graaff et al. conducted a survey among 931 women and showed that 16% of respondents declared time lost in education due to endometriosis symptoms, of which 35% delayed their final exams and 15% had to drop out of college. One in two surveyed women reported that the disease had adversely affected their professional work, of which 48% had reduced their working hours, 11% had lost their jobs and 7% had changed jobs [3]. All of the above contribute to high costs for the government and the healthcare system [13].

Among our study population, dysmenorrhea and infertility were the most frequently identified symptoms. According to scientific reports, painful menstruation is the most commonly reported symptom, affecting every second patient with endometriosis [14]. Despite it being so burdensome, it is often underestimated. De Graaff et al. showed that patients were consulted by approximately three different gynecologists before they received the proper diagnosis [3]. A study on a large population of female patients from 10 countries reported a diagnostic delay of 6.7 years. It took an average of 8 years in the UK [15], and 10.4 years in Germany and Austria [9]. In our study, the time from the onset of symptoms to the diagnosis of endometriosis averaged 5.7 years (median of 5 years), which is consistent with the reports of other authors [3,15].

Alongside dysmenorrhea, infertility was the second most commonly associated symptom. Eisenberg et al. published a large study based on a computer database covering a quarter of Israel′s population. It showed the incidence of infertility among women with endometriosis at the level of 36.9%. Due to difficulties in conceiving, those patients had appointments more often. The same study reported that 70% of women with endometriosis saw their primary care physician seven times per year or more, and one in five visited a gynecologist 5× per year or more [16].

According to our study population, painful defecation, dysuria and sciatica were the least frequently indicated as associated with endometriosis. Pain during bowel movements may be perceived by patients as a gastrointestinal complaint rather than a symptom that will be resolved by gynecologists. Singh et al. analyzed the prevalence of endometriosis symptoms in the Canadian population and revealed that half of the patients had pain during defecation, and 40% had complaints related to urination [10]. When taking medical history, the patient may not mention such complaints herself, so it is crucial to investigate any defecation or micturition discomfort. A comparative analysis revealed that more women with versus without DxE experienced menstrual pelvic pain or cramping (70.3% vs. 50.7%), non-menstrual pelvic pain or cramping (49.5% vs. 18.7%), dyspareunia (52.5% vs. 28.0%) and infertility (22.3% vs. 6.3%) [10]. All of the above symptoms were statistically more common among respondents with DxE than non-DxE in our study.

According to DiVasta et al. the analysis of the spectrum of symptoms of endometriosis between adolescents and adults pointed to slight differences in disease presentation. Younger patients significantly more often reported nausea accompanying pain (69% vs. 53%; *p* = 0.01). The authors concluded that, given the general population of young women, the combination of pelvic pain with nausea during menstruation should raise the possibility of endometriosis and could be considered as a marker [17]. In our study, adolescents comprised a small group, and we did not ask about the presence of nausea during menstruation.

Research on markers to aid in diagnosis has been ongoing for years [18]. The variety of symptoms of the disease makes the diagnosis extremely difficult, even for an experienced doctor. The most common misdiagnoses are chronic pelvic pain syndrome, bleeding disorders, irritable bowel syndrome (IBS), gastrointestinal intolerances, psychosexual complaints, irritable bladder, pelvic inflammatory disease (PID), appendicitis and idiopathic sterility. As many as 73.4% of the examined patients received at least one misdiagnosis, which prolonged the diagnostic process and led to misplaced pharmacotherapy [9].

The strength of our study was the broad population of female respondents surveyed. However, CAWI-based surveys, such as ours, have some limitations. They do not reach certain types of respondents (those without Internet access could not complete the survey) and it lacks verification tools (to avoid one person submitting multiple responses). All symptoms included in the survey were related to endometriosis, and women with this disease could have had all of them, causing bias. Moreover, respondents interested in the problem of endometriosis might have added additional bias to the study—since they were interested, they were more likely to participate than those who were not interested. Nevertheless, even if they were interested, their knowledge was not satisfactory. In addition, we did not ask about the stage of the disease, but as it is known from the literature, it does not corelate with disease symptoms [4].

Shah et al. concluded that by increasing public awareness of endometriosis, we could enable early diagnosis and alleviate the social isolation reported by patients with endometriosis [19]. The analysis of the current state of knowledge on a large sample of Polish women can be a good starting point for further educational activities to improve the diagnosis of the disease, shorten the time from the onset of symptoms to obtaining a diagnosis and implementing appropriate treatment.

## 5. Conclusions

Our study evaluated the level of knowledge of endometriosis symptoms to better understand the public awareness of the disease. The results showed moderate to low levels of knowledge about endometriosis symptoms. Since the search for a non-invasive diagnostic marker of the disease has been conducted for many years without success so far, it seems that the clinical presentation and reported symptoms might be crucial for decreasing the delay in diagnosis.

As the Internet was the most frequently quoted as the main source of knowledge for the study population, it could be the most appropriate place to spread reliable information regarding endometriosis and serve as an online screening test, based on specially designed applications.

## Figures and Tables

**Table 1:** Demographics of the study population.

Age	Women DxE * *n* (%)	Women Non-DxE *n* (%)	Overall
≤25	45 (1.4%)	857 (25.8%)	902 (27.2%)
26-30	120 (3.6%)	1126 (33.9%)	1246 (37.5%)
31–35	101 (3.0%)	645 (19.4%)	746 (22.4%)
36–40	41 (1.2%)	240 (7.3%)	281 (8.5%)
≥41	21 (0.6%)	123 (3.8%)	144 (4.4%)
**Place of Residence**			
City over 500 k	119 (3.6%)	1017 (30.6%)	1136 (34.2%)
City between 100–500 k	57 (1.7%)	593 (17.9%)	650 (19.6%)
City below 100 k	83 (2.5%)	744 (22.4%)	827 (24.9%)
Countryside	69 (2.1%)	637 (19.2%)	706 (21.3%)
**Education**			
Higher	189 (5.7%)	1455 (43.8%)	1644 (49.5%)
Secondary	73 (2.2%)	612 (18.5%)	685 (20.7%)
Primary	66 (2.0%)	922 (27.8%)	988 (29.8%)
**Medical Profession**			
Yes	35 (1.1%)	513 (15.5%)	548 (16.6%)
No	293 (8.8%)	2476 (74.6%)	2769 (83.4%)

* DxE—respondents with endometriosis.

**Table 2:** Identification of symptoms according to respondents with medical occupations (*n* = 548) and without medical occupations (*n* = 2769).

Symptom	Women with Medical Occupations; Sum of Responses: Strongly Associate (5) and Moderately Associate (4)	Women without Medical Occupations; Sum of Responses: Strongly Associate (5) and Moderately Associate (4)	*p*-Value
Infertility	94.2%	89.2%	*p* = 0.0009
Dysmenorrhea	87.5%	84.8%	*p* = 0.0921
Dyspareunia	72.1%	55.8%	*p* < 0.0001
CPP *	69.5%	61.4%	*p* = 0.0030
Diarrhea/constipation	48.7%	37.8%	*p* < 0.0001
Sacral pain	56.4%	42.7%	*p* < 0.0001
Painful defecation	26.2%	18.0%	*p* < 0.0001
Dysuria	27.6%	18.8%	*p* < 0.0001
Sciatica	20.6%	19.9%	*p* < 0.0001

* CPP—chronic pelvic pain not related to menstruation.

**Table 3:** Identification of symptoms according to respondents with endometriosis (DxE) and without endometriosis (Non-DxE).

Symptom		Strongly Disagree (1)	Disagree (2)	Neutral (3)	Agree (4)	Strongly Agree (5)	*p* Value
Infertility	DxE Non-DxE	1.2% 3.2%	0.6% 1.4%	2.4% 6.0%	11.0% 14.6%	84.8% 74.8%	*p* < 0.0001
Dysmenorrhea	DxE Non-DxE	0.6% 4.5%	0.9% 3.0%	2.4% 8.4%	10.1% 18.0%	86.0% 66.1%	*p* < 0.0001
Dyspareunia	DxE Non-DxE	7.6% 13.6%	3.0% 8.5%	14.0% 21.2%	22.3% 25.4%	53.1% 31.3%	*p* < 0.0001
CPP *	DxE Non-DxE	6.4% 9.1%	6.7% 9.8%	12.5% 19.7%	15.5% 24.5%	58.9% 36.9%	*p* < 0.0001
Diarrhea/Constipation	DxE Non-DxE	13.7% 28.7%	5.8% 13.9%	11.9% 21.0%	20.1% 17.9%	48.5% 18.5%	*p* < 0.0001
Sacral Pain	DxE Non-DxE	11.6% 18.9%	7.4% 13.0%	13.7% 25.6%	21.3% 24.1%	46.0% 18.4%	*p* < 0.0001
Painful Defecation	DxE Non-DxE	24.1% 45.0%	10.7% 20.1%	14.6% 19.0%	19.2% 8.7%	31.4% 7.2%	*p* < 0.0001
Dysuria	DxE Non-DxE	27.1% 40.0%	13.7% 20.7%	19.2% 21.2%	15.6% 10.9%	24.4% 7.2%	*p* < 0.0001
Sciatica	DxE Non-DxE	33.2% 42.0%	12.5% 18.9%	17.7% 20.9%	11.0% 10.2%	25.6% 8.0%	*p* < 0.0001

* CPP—chronic pelvic pain not related to menstruation.

## References

[1] Soliman A.M., Yang H., Du E.X., Kelley C., Winkel C. (2016). The direct and indirect costs associated with endometriosis: A systematic literature review. Hum. Reprod..

[2] Gruppo Italiano per lo Studio dell’Endometriosi (2001). Relationship between stage, site and morphological characteristics of pelvic endometriosis and pain. Hum. Reprod..

[3] De Graaff A.A., D’Hooghe T.M., Dunselman G.A., Dirksen C.D., Hummelshoj L., Simoens S. (2013). The significant effect of endometriosis on physical, mental and social wellbeing: Results from an international cross-sectional survey. Hum. Reprod..

[4] Warzecha D., Szymusik I., Wielgos M., Pietrzak B. (2020). The impact of endometriosis on the quality of life and the incidence of depression-A cohort study. Int. J. Environ. Res. Public Health.

[5] Sinaii N., Cleary S.D., Ballweg M.L., Nieman L.K., Stratton P. (2002). High rates of autoimmune and endocrine disorders, fibromyalgia, chronic fatigue syndrome and atopic diseases among women with endometriosis: A survey analysis. Hum. Reprod..

[6] Della Corte L., Di Filippo C., Gabrielli O., Reppuccia S., La Rosa V.L., Ragusa R., Fichera M., Commodari E., Bifulco G., Giampaolino P. (2020). The burden of endometriosis on women’s lifespan: A narrative overview on quality of life and psychosocial wellbeing. Int. J. Environ. Res. Public Health.

[7] Fourquet J., Báez L., Figueroa M., Iriarte R.I., Flores I. (2011). Quantification of the impact of endometriosis symptoms on health-related quality of life and work productivity. Fertil. Steril..

[8] Nawrocka-Rutkowska J., Szydłowska I., Rył A., Ciećwież S., Ptak M., Starczewski A. (2021). Evaluation of the diagnostic accuracy of the interview and physical examination in the diagnosis of endometriosis as the cause of chronic pelvic pain. Int. J. Environ. Res. Public Health.

[9] Hudelist G., Fritzer N., Thomas A., Niehues C., Oppelt P., Haas D., Salzer H., Tammaa A. (2012). Diagnostic delay for endometriosis in Austria and Germany: Causes and possible consequences. Hum. Reprod..

[10] Singh S., Soliman A.M., Rahal Y., Robert C., Defoy I., Nisbet P., Leyland N. (2020). Prevalence, Symptomatic Burden, and Diagnosis of Endometriosis in Canada: Cross-Sectional Survey of 30 000 Women. J. Obstet. Gynaecol. Can..

[11] Horne A.W., Saunders P.T.K., Abokhrais I.M., Hogg L. (2017). Top ten endometriosis research priorities in the UK and Ireland. Lancet.

[12] Moradi M., Parker M., Sneddon A., Lopez V., Ellwood D. (2014). Impact of endometriosis on women’s lives: A qualitative study. BMC Womens Health.

[13] Klein S., D’Hooghe T., Meuleman C., Dirksen C., Dunselman G., Simoens S. (2014). What is the societal burden of endometriosis-associated symptoms? A prospective Belgian study. Reprod. Biomed. Online.

[14] Gałczyński K., Jóźwik M., Lewkowicz D., Semczuk-Sikora A., Semczuk A. (2019). Ovarian endometrioma—A possible finding in adolescent girls and young women: A mini-review. J. Ovarian Res..

[15] Ghai V., Jan H., Shakir F., Haines P., Kent A. (2020). Diagnostic delay for superficial and deep endometriosis in the United Kingdom. J. Obstet. Gynaecol..

[16] Eisenberg V.H., Weil C., Chodick G., Shalev V. (2018). Epidemiology of endometriosis: A large population-based database study from a healthcare provider with 2 million members. Bjog.

[17] DiVasta A.D., Vitonis A.F., Laufer M.R., Missmer S.A. (2018). Spectrum of symptoms in women diagnosed with endometriosis during adolescence vs adulthood. Am. J. Obstet. Gynecol..

[18] Toczek J., Jastrzębska-Stojko Ż., Stojko R., Drosdzol-Cop A. (2021). Endometriosis: New perspective for the diagnosis of certain cytokines in women and adolescent girls, as well as the progression of disease outgrowth: A Systematic Review. Int. J. Environ. Res. Public Health.

[19] Shah D.K., Moravek M.B., Vahratian A., Dalton V.K., Lebovic D.I. (2010). Public perceptions of endometriosis: Perspectives from both genders. Acta Obstet. Gynecol. Scand..

